# *In vitro* behaviour of endothelial cells on a titanium surface

**DOI:** 10.1186/1746-160X-4-14

**Published:** 2008-07-23

**Authors:** Ana Cristina Breithaupt-Faloppa, Wothan Tavares de Lima, Ricardo Martins Oliveira-Filho, Johannes Kleinheinz

**Affiliations:** 1 Department of Pharmacology, Institute of Biomedical Sciences, University of Sao Paulo, Brazil; 2 Department for Cranio-Maxillofacial Surgery, University Hospital Muenster, Germany

## Abstract

**Background:**

Endothelial cells play an important role in the delivery of cells to the inflammation site, chemotaxis, cell adhesion and extravasation. Implantation of a foreign material into the human body determines inflammatory and repair reactions, involving different cell types with a plethora of released chemical mediators. The evaluation of the interaction of endothelial cells and implanted materials must take into account other parameters in addition to the analysis of maintenance of cell viability.

**Methods:**

In the present investigation, we examined the behavior of human umbilical vein endothelial cells (HUVECs) harvested on titanium (Ti), using histological and immunohistochemical methods. The cells, after two passages, were seeded in a standard density on commercially plate-shaped titanium pieces, and maintained for 1, 7 or 14 days.

**Results:**

After 14 days, we could observe a confluent monolayer of endothelial cells (ECs) on the titanium surface. Upon one-day Ti/cell contact the expression of fibronectin was predominantly cytoplasmatic and stronger than on the control surface. It was observed strong and uniform cell expression along the time of α5β1 integrin on the cells in contact with titanium.

**Conclusion:**

The attachment of ECs on titanium was found to be related to cellular-derived fibronectin and the binding to its specific receptor, the α5β1 integrin. It was observed that titanium effectively serves as a suitable substrate for endothelial cell attachment, growth and proliferation. However, upon a 7-day contact with Ti, the Weibel-Palade bodies appeared to be not fully processed and exhibited an anomalous morphology, with corresponding alterations of PECAM-1 localization.

## Background

Since the discovery of endothelial-derived relaxing factor (EDRF) by Furchgott & Zawadzki [1], in 1980, endothelial cells (ECs) have been recognized to be involved in vascular homeostasis, angiogenesis and repair of injured tissues. ECs play an important role in the trafficking of cells from bloodstream towards an inflammatory site, chemotaxis, cell adhesion and extravasation [2]. Factors released by ECs mediate the control of vascular tonus, thrombogenesis and fibrinolysis, and platelet activities [3]. Besides, by interacting with cytokines and leukocytes, ECs orchestrate the inflammatory process [4], a fact involved with the complex phenomena observed at the host implant interface. ECs produce and store the haemosthatic protein von Willebrand factor (vWf) into granules named Weibel Palade bodies (WPBs), that are secretory organelles. They thus provide a readily releasable pool of extracellular VWF as well as placing P-selectin on the plasma membrane whereby it can recruit leukocytes and thus play a role in the initiation of inflammation [5].

Implantation of a foreign material into the intimity of human tissues triggers a typical inflammatory response followed by tissue repair. After implanted, the material will determine the clinical outcome and will have an influence on the implantation bed, triggering cellular and non-cellular responses [6]. Metals and alloys are the most common materials used as surgical implants in order to replace mineralised structures [7,8]. In particular, titanium alloys show properties which render them suitable substrates for surgical implant [6,8]. Moreover, the high degree of biocompatibility of titanium and its alloys is intimately related to the passively formed oxide film on the metallic surface [9,10].

Noteworthy, the evaluation of the interaction of cells and implanted materials must take into account other parameters in addition to the analysis of maintenance of cell viability. Indeed, the interaction of implants with host cells, and in particular with endothelial cells, might cause activation of adhesion molecules culminating with cytokine generation [2]. In fact, the degree of expression of adhesion molecules on the surface of human ECs depends on the response of the cells against the implanted material [11].

PECAM-1 is a cell-cell junction molecule that establishes homophilic binding between neighboring ECs [12]. PECAM-1 interacts with the underlying cytoskeleton and regulates F-actin assembly at the cell periphery in association with changes in cell shape and spreading [13]. The mechanism of endothelial cell adhesion to substrates involves integrins expression, thence connecting extracellular matrix (ECM) with the cytoskeleton [14,15]. Integrins are also considered to be the main receptors of ECM proteins, such as fibronectin, laminin, collagens, and vitronectin. Altogether, these proteins constitute the main mediators of cell-ECM adhesion [14].

There is evidence that upon binding to an ECM protein (e.g. fibronectin), a number of integrins mediate cellular signaling and functions. It was shown that α5β1 integrin, a receptor for both fibronectin (FN) and vitronectin (VN), and αvβ3 integrin, a VN receptor, both play a role in angiogenesis [16]. Therefore, the success of vasculogenesis and angiogenesis depends on FN [17,18] and its main receptor, the α5β1 integrin [19]. Upon wound repair, angiogenetic mechanisms are called into play leading to generation of new capillary blood vessels [20]. Accordingly, angiogenesis is of pivotal importance during the initial healing process, and thus the characterization of the cellular responses involved in angiogenesis and bone formation adjacent to the implants is critical to understanding and promoting implant biocompatibility and improving stable fixation of implants [21].

Tissue repair around an implanted piece of Ti depends crucially on osseous integration and angiogenesis. Though a huge deal of information exist about bone modifications in this situation, the interaction of this metal with endothelial cells is not completely understood. Being so, in this study we investigated the behavior of ECs in culture on Ti plates and assessed the protein expression and cell adhesion, in an attempt to better understand the reasons why Ti-made implant materials achieve successful clinical application.

## Materials and methods

The experimental design was approved by the Ethics Committee, Faculty of Medicine, University of Münster. The material analyzed in this study was commercially available, plate-shaped pieces of pure titanium (Ti). As control surfaces, we used round plastic coverslips (Thermanox^®^, Nunc, USA) coated with gelatin (Sigma, USA).

### Antibodies

All antibodies were used as purified IgGs. Monoclonal antibodies: anti-human CD-31 (PECAM-1), anti-human vinculin (Sigma, USA); anti-human fibronectin receptor (Takara Biomedicals, Japan); anti-human vitronectin, anti-human vitronectin receptor and anti-human VE-cadherin (Chemicon International, USA); anti-human α-smooth muscle actin (ICN Biomedicals, USA). Polyclonal antibodies: anti-human von Willebrand factor (vWf)/factor VIII (ICN Biomedicals, USA) and anti-fibronectin (Biotrend, Germany). Second antibodies: alexa fluor 488 goat anti-mouse, and anti-rabbit.

### Cultures of human umbilical vein endothelial cells (HUVECs)

The endothelial cells were isolated from umbilical cord veins essentially as described by Marin *et al*. [22], according to the method of Jaffe *et al*. [23]. Cells were pooled and established as primary cultures seeded on 0,5% gelatin-coated (gelatin 2% solution) tissue culture dishes in medium 199 enriched with 20% heat-inactivated fetal bovine serum, 5 μg/ml amphotericin B, 200 U/ml penicillin, 200 μg/ml streptomycin, 1% endothelial cell growth supplement, and 0,1% heparin. Cultures were carried out at 37°C in a humidified atmosphere with 5% CO_2_; the culture medium was changed every other day. The cultures were serially passaged by incubating confluent cells in 0.05% trypsin/0.02% EDTA solution and replating them at a 1:2 ratio. Second passage cells were taken for the experiments after being identified as endothelial cells by staining with a panel of endothelial-specific antibodies (anti-vWF, anti-CD-31 [PECAM-1]) and by a negative staining to anti-α-smooth muscle actin.

### Contact assays

Detachment of cells was performed by trypsinisation during 1 min; the reaction was stopped by dilution with enriched medium and the resulting suspensions were centrifuged (1,200 rpm for 3 min). The pellet was resuspended in growth medium. Cell numbers were determined with a cell counter. Titanium plates (1 cm^2^) were placed onto the bottoms of 24-well plates, and the cells were seeded in every well at a density of 8 × 10^4 ^cells/cm^2^. As control surfaces, we used round pieces (plastic coverslips) with the same dimensions, coated with gelatin and seeded with the same concentration of cells. The experiments were carried out during 1, 7 or 14 days and the media were changed every other day.

### Immunohistochemistry

At the end of every culture period, both cell substrates were processed with a fluorescent staining method and studied with a confocal microscope. For this purpose, the substrates inside the wells were rinsed with PBS and fixed in methanol for 20 minutes at -4°C. Non-specific sites were then blocked by incubation during 15 minutes at room temperature with Tris-buffered saline/Tween 20^® ^(TBST) containing 0.5% bovine serum albumin. Afterwards, the substrates were incubated with the primary antibodies during 1 h at 37°C, rinsed three times with TBST and then incubated with the correspondent alexa fluor 488 second antibody for 1 h at 37°C. Negative controls were prepared using incubations with primary antibodies-free saline. Images of the stained probes were captured using a confocal microscope (Zeiss Axiovert).

### Scanning electron microscopy

The titanium plates were fixed with 2.5% glutaraldehyde and then dehydrated using a graded ethanol series. The process was completed by critical point drying using CO_2 _and a thin layer of gold was sputter-coated onto the plates prior to examination. The images of the surfaces were captured using a Philips PSEM500× microscope.

### Cell countings

After the corresponding culture periods, titanium and control pieces were rinsed with PBS to eliminate unattached cells. The adherent cells were removed by incubation with trypsin/EDTA for 2 minutes at 37°C. Trypsin action was stopped by dilution with complete growth medium at room temperature and 100 μl-samples of the resulting cell suspensions were counted using a cell counter (Casy^®^1, Schärfe System, Germany). Results were expressed as the number of adherent cells per sample of titanium or control and were analyzed by one-way analysis of variance and the Tukey-Kramer multiple comparisons test. A 2.01 version GraphPad InStat™ software was used for this purpose. When appropriate, the Student's *t *test was also used.

## Results

### Material characterization

**Figure 1:**
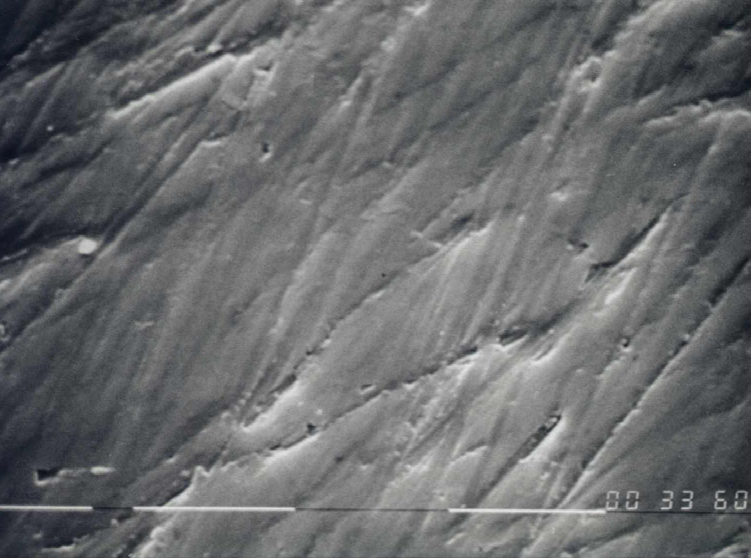
Scanning electron microscopy aspect of titanium plate without cells (2500 ×).

Images of titanium plates showed the surface topography and revealed the presence of regular parallel grooves (Figure 1).

### Cell morphology

**Figure 2:**
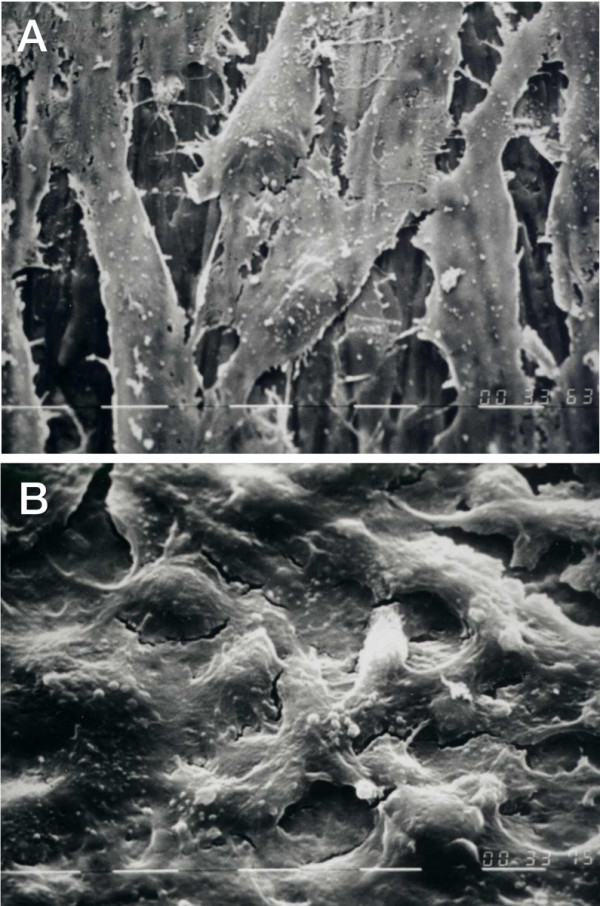
Scanning electron microscopy image of endothelial cells attached to the titanium plate after 1 day of culture (A) and after 14 days (B) (1250 ×).

The morphological observations indicated that the cells seeded on titanium seem to conform to the material surface and to be flat, elongated and oriented along the titanium grooves (Figure 2A). After 7 days we could observe the presence of increased number of cells; after 14 days there was a confluent and dense cell layer (Figure 2B). Cells over the control surface maintained their morphological patterns without any significant volume variation or other modifications.

### Titanium assays and immunohistochemistry

HUVECs adhered to the surface, spread and proliferated and within 24 hours started forming a subconfluent monolayer. On titanium and on control surfaces it could be noticed that the cells proliferated and reached confluence, throughout the experimental period.

**Figure 3:**
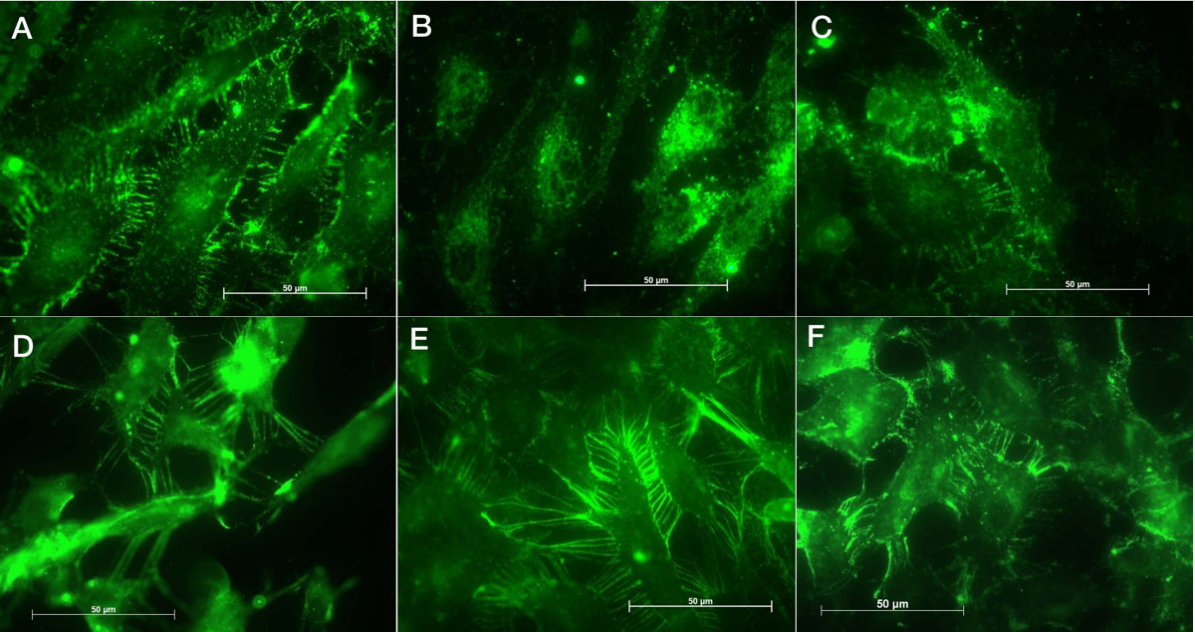
Images of the immunohistochemical assays: PECAM-1 on endothelial cells attached to titanium after (A) 24 hours, (B) 7 days and (C) 14 days, and to control surface after (D) 24 hours, (E) 7 days and (F) 14 days.

**Figure 4:**
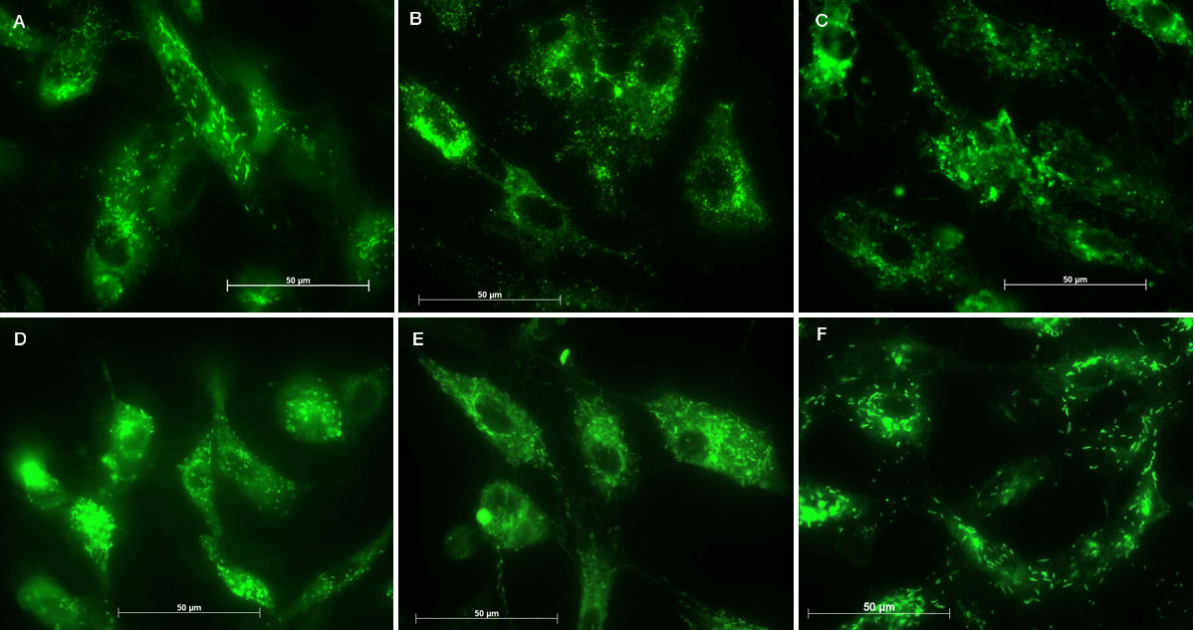
Images of the immunohistochemical assays: von Willebrand factor on endothelial cells attached to titanium after (A) 24 hours, (B) 7 days and (C) 14 days, and to control surface after (D) 24 hours, (E) 7 days and (F) 14 days.

Stainings with antibodies anti- PECAM-1 and anti-vWf were performed to confirm the conservation of the endothelial characteristics. There was a mild up to strong expression of PECAM-1 by the cells and no expression was evidenced on the cellular processes after 7 days (Figures 3A, B and 3C). Conversely, on the control surfaces the CD-31 expression did not vary during the studied periods (Figures 3D, E and 3F). Cells tested for vWf showed the presence of this factor on titanium and on control surfaces, but on titanium after 7 and 14 days we could not observe well-defined granules in the cells and after 7 days they are distributed on the perinuclear region (Figure 4B and 4C). Conversely, on control surfaces the cells presented well-defined vWf granules uniformly distributed in the cells in all studied periods (Figures 4D, E and 4F).

**Figure 5:**
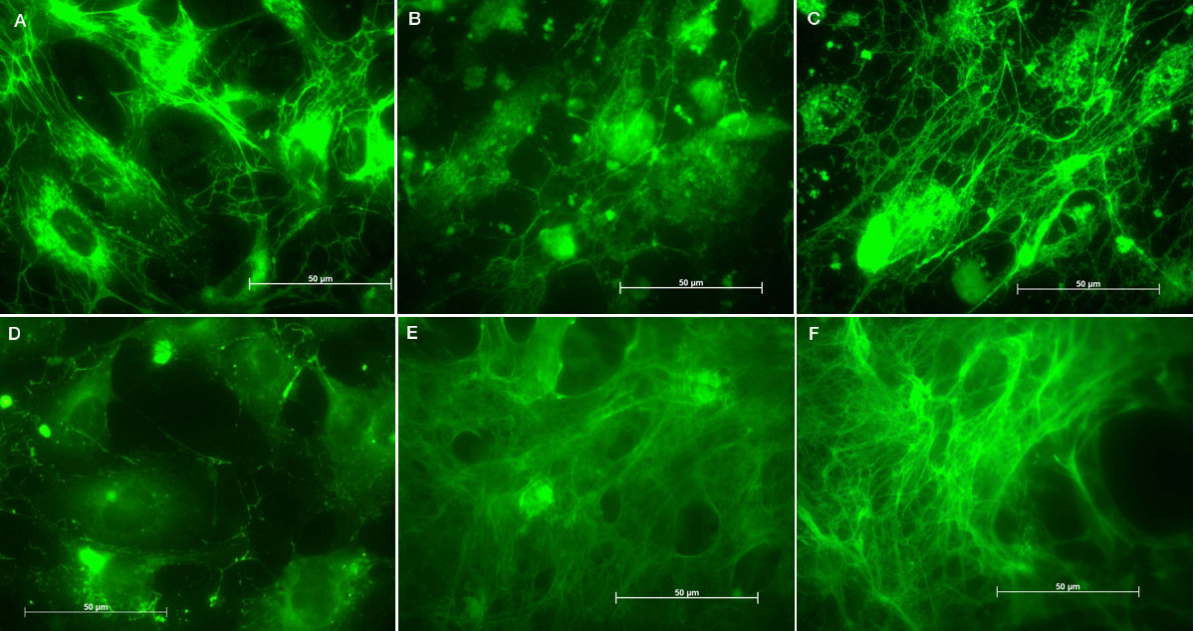
Images of the immunohistochemical assays: fibronectin on endothelial cells on titanium after (A) 24 hours, (B) 7 days and (C) 14 days and on control surface after (D) 24 hours, (E) 7 days and (F) 14 days.

The cells were also studied for the presence of extracellular matrix proteins, fibronectin and vitronectin. The results on both surfaces showed a strong positive reaction for fibronectin with a progressively intensity increase. Upon one-day Ti/cell contact the expression of fibronectin was predominantly cytoplasmatic and stronger than on the control surface (Figure 5A and 5D). After 7 and 14 days we observed that fibronectin was predominantly extracellular on both surfaces (Figures 5B, C, E and 5F). In contrast, no positive responses were obtained with the specific anti-vitronectin antibody.

Assays for VE-cadherin expression resulted negative, both on control and titanium surfaces for any experimental period studied.

**Figure 6:**
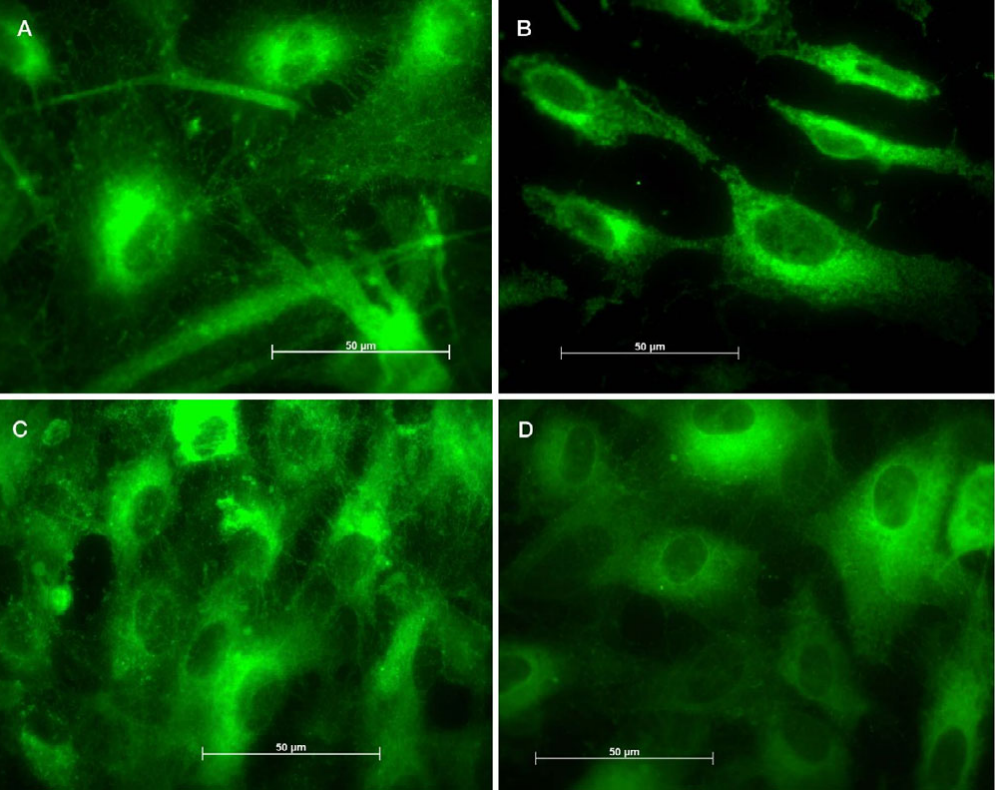
Images of the immunohistochemical assays: α5β1 integrin expression on endothelial cells attached to titanium after (A) 24 hours, (B) 7 days and (C) 14 days and to control surface (D).

**Figure 7:**
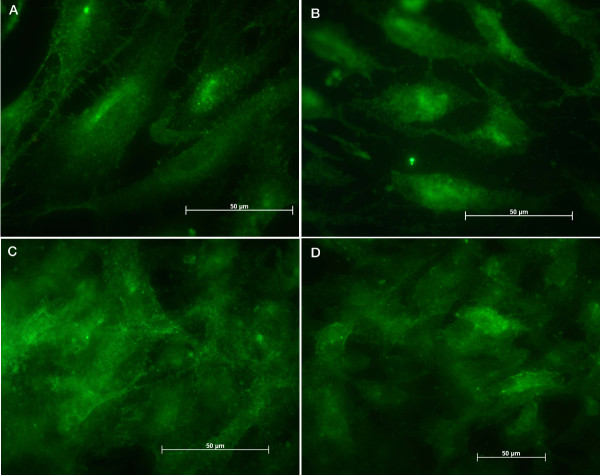
Images of the immunohistochemical assays: α(v)β3 integrin expression on endothelial cells attached to titanium after (A) 24 hours, (B) 7 days and (C) 14 days and to control surface (D).

The assays for integrins α5β1 (Figure 6) and α(v)β3 (Figure 7) revealed strong and uniform cell expression along the time, with a stronger reaction for α5β1 on the cells in contact with titanium or control surfaces.

Similar testings were done for vinculin; the staining was positive and did not significantly vary along the time. The subcellular distribution of this protein, along the Ti/cell contact time, was similar to that of the α5β1 integrin found after 7 days.

### Cell countings

**Figure 8:**
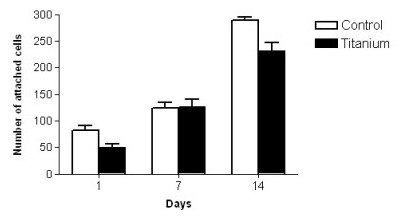
**Attachment of HUVECs on titanium and on control surfaces after the studied periods.** Values are mean ± SEM.

Figure 8 shows the number of cells attached to titanium and control surfaces after the different growing periods (1, 7 or 14 days). Overall, there was an increasing number of cells adhered to the substrates along the time, without significant difference between titanium and control surface.

## Discussion

Besides requiring invasion by endothelial cells (ECs), angiogenesis depends upon localized proteolytic modifications of the extracellular matrix (ECM) and/or a substrate to which ECs can adhere, migrate upon, proliferate within, and eventually differentiate into a mature EC phenotype. The physical characteristics as well as the composition of the material must be suitable for cell adherence, because focal adhesions can only be formed and maintained when the material sustains cell adhesion. The cellular response to a foreign material is dictated largely by the surface properties of the material to which the cells contact. A better understanding of the role of occurring matrix in the various steps of angiogenesis would conceivably contribute to the intelligent design of tissue-engineered constructs where angiogenesis is critical for tissue repair and restoration [24].

Results from van Kooten et al. [25] showed that HUVECs adhere to metal surfaces and start forming a subconfluent monolayer within 3 days, and that focal contacts are present after 3 days of adhesion, with the cells still displaying their endothelial phenotype. It has been demonstrated that various specific proteins adsorb onto the scaffolds used for culture from serum-containing medium *in vitro*, and that cells use specific integrin receptors to bind to these proteins [26].

Tissue repair around an implanted piece of Ti depends crucially on osseous integration and angiogenesis. Though a huge deal of information exist about bone modifications in this situation, the interaction of endothelial cells with Ti is not completely understood. A permanent state of oxidative stress appears to exist in endothelial cells grown in direct contact with Ti surfaces [27]. These authors showed that HUVECs adhere to Ti *in vitro *and a complete coverage of an EC layer was obtained without any coating or surface treatment.

Cell adhesion to implants *in vivo *and to culture surfaces *in vitro *is typically dependent upon surface-adsorbed fibronectin and vitronectin [28]. After 1 day on titanium (see Fig. 5) it could be observed a strong cytoplasmatic pool of fibronectin on ECs and a growing extracellular mesh. Conversely, on control surfaces that fibronectin pool was not observed. After 7 days this protein was found over both surfaces with a more dense mesh on the control surfaces. The cells produced and released fibronectin in order to use this protein as a substrate for attachment, with a stronger presence in the cells after 1 day on titanium. On control surfaces the initial attachment was most conceivably based on the gelatin coat. Although it has been stated that over time the adhesion may be progressively more dependent on vitronectin in view of the larger amounts, or the preferential binding to this molecule [29,30], our data do not support this view, since our EC vitronectin stainings failed to demonstrate any positive answer either on titanium or control surfaces. Our findings showed that ECs produced and eliminated endogenous fibronectin, using this protein to attach to the surface through integrins (namely, αvβ1 and αvβ3) which are expressed when ECs were seeded on titanium. The observed expression of αvβ3 was weaker in comparison to that of α5β1 (Figs. 6 and 7).

The EC morphology on titanium plates was not altered. However, WPBs were not well-defined after 7 and 14 days and they were distributed on the perinuclear region. Some authors report that only fully processed, substantially polymerized and functionally mature vWf is stored in the WPBs, together with its propeptide [31]. Interestingly, it is established that HUVECs poses two different populations of WPBs, that are differentiated at cellular level by their distribution, the newly formed ones being immature and located in the perinuclear region [5]. Thus it is conceivable to infer that on titanium, after 7 and 14 days, the WPB on HUVECs were immature in nature as judged by their distribution (Fig. 4) whereas the mature granules were secreted. On the other hand, the presence and the distribution of the WPBs on HUVECs on control surfaces were uniform in all studied periods.

Since PECAM-1-mediated pathway could be involved in the observed differences in cell behavior, the expression level of PECAM-1 on titanium was analyzed. CD-31 (PECAM-1) was expressed mildly up to strongly by the cells on Ti, but we could not observe the expressed CD-31 on the cellular processes after 7 days (Fig. 3). Since this was not observed with control surfaces, those cell contact alterations might well be due to an effect of titanium on HUVECs.

Our results did not show the presence of VE-cadherin within junctions in all periods studied neither in contact with titanium nor with the control surface. This could be an evidence of a lack of some stimulus (i) for this molecule to trigger its participation on the junctions.

## Conclusion

In conclusion, our data indicated that the attachment of ECs on titanium could be related to cellular-derived fibronectin and the binding to its specific receptor, the α5β1 integrin. It was observed that titanium effectively serves as a suitable substrate for endothelial cell attachment, growth and proliferation in the initial phase. It is suggested to describe this feature of titanium if not angiogenic then at least angio-conductive. However, upon a 7-day contact with Ti the Weibel-Palade bodies were found to be not fully processed and with altered morphology, which corresponding alterations of PECAM-1 localization. In fact that the successful Ti devices implantation coexists with the microscopical subclinical adverse effects is, at the present, unresolved.

## Authors' contributions

ACB–F performed all experiments under the supervision of JK. ACB–F drafted the manuscript and was advised by RMO–F and WTdL. All authors read and approved the final manuscript.
